# Identification and quantification of pathogenic helminth eggs using a digital image system

**DOI:** 10.1016/j.exppara.2016.04.016

**Published:** 2016-07

**Authors:** B. Jiménez, C. Maya, G. Velásquez, F. Torner, F. Arambula, J.A. Barrios, M. Velasco

**Affiliations:** a Instituto de Ingeniería, UNAM, P.O. Box 70-186, México, D.F., 04510, Mexico; b Centro de Ciencias Aplicadas y Desarrollo Tecnológico, UNAM, P.O. Box 70-186, México, D.F., 04510, Mexico

**Keywords:** Digital image algorithm, Helminth eggs, Identification, Quantification, Automated system, Wastewater

## Abstract

A system was developed to identify and quantify up to seven species of helminth eggs (*Ascaris lumbricoides* -fertile and unfertile eggs-, Trichuris trichiura, Toxocara canis, Taenia saginata, Hymenolepis nana, Hymenolepis diminuta, and Schistosoma mansoni) in wastewater using different image processing tools and pattern recognition algorithms. The system was developed in three stages. Version one was used to explore the viability of the concept of identifying helminth eggs through an image processing system, while versions 2 and 3 were used to improve its efficiency. The system development was based on the analysis of different properties of helminth eggs in order to discriminate them from other objects in samples processed using the conventional United States Environmental Protection Agency (US EPA) technique to quantify helminth eggs. The system was tested, in its three stages, considering two parameters: specificity (capacity to discriminate between species of helminth eggs and other objects) and sensitivity (capacity to correctly classify and identify the different species of helminth eggs). The final version showed a specificity of 99% while the sensitivity varied between 80 and 90%, depending on the total suspended solids content of the wastewater samples. To achieve such values in samples with total suspended solids (TSS) above 150 mg/L, it is recommended to dilute the concentrated sediment just before taking the images under the microscope. The system allows the helminth eggs most commonly found in wastewater to be reliably and uniformly detected and quantified. In addition, it provides the total number of eggs as well as the individual number by species, and for *Ascaris lumbricoides* it differentiates whether or not the egg is fertile. The system only requires basically trained technicians to prepare the samples, as for visual identification there is no need for highly trained personnel. The time required to analyze each image is less than a minute. This system could be used in central analytical laboratories providing a remote analysis service.

## Highlights

• The system identifies and quantifies seven species of helminth eggs.
• The system shows a specificity of 99% and a sensitivity between 80 and 90%.
• The time required to analyze each image is less than a minute.
• The system reduces the need for highly trained personnel for the identification of helminth eggs.

## Graphical abstract

**Figure:**
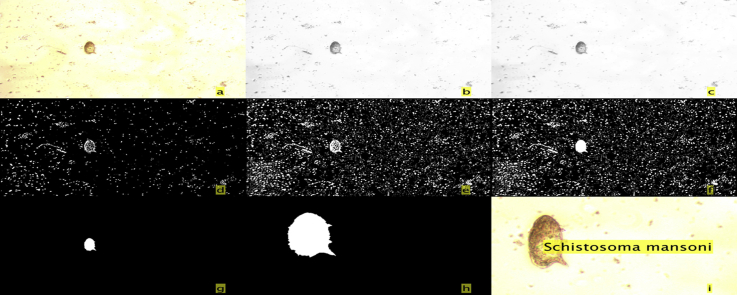

## 1 Introduction

Wastewater reuse in agriculture has a long history. In some countries, it is practiced with highly treated wastewater, while in many developing countries, where sanitation coverage is still poor, low quality water is used. It results from high water demand for irrigation (81% of the total water extracted for use in developing countries compared to only 45% in developed ones; Blumenthal et al., 2001) and the lack of access to water with high quality. In many regions polluted water has become a key resource for food production and to improve economy. Water reuse for irrigation saves significant volumes of fresh water, provides nutrients to soil, reducing or eliminating the need for chemical fertilizers, contributes to the expansion of agricultural land in arid and semi-arid areas, increases income for farmers, and is a relatively cheap disposal method for wastewater avoiding the pollution of other surface water bodies. Both the availability of water and the nutrients contained in the wastewater used for irrigation increase soil fertility and crop yield and enable the cultivation of produce with higher profitability.

There are no formal statistics on the reuse of low quality water for irrigation in agricultural fields; however, it is estimated that at least 20 million ha in 50 developing countries (around 10% of their total irrigated land; UN, 2003) are watered this way. In the Tula Valley, Mexico, alone, 90,000 ha use around 50 m^3^/s of untreated wastewater produced in Mexico City. Examples of the reuse of treated wastewater in developing countries are also available. For instance in Latin America, in Mendoza, Argentina 129,600 m^3^/d of effluent from stabilization ponds are used for the irrigation of 1900 ha; in Chile, the Maipo and Maipocho and the Santiago de Chile regions have, respectively, 130,000 ha and 110,000 ha reusing treated wastewater (Navarro et al., 2015). In some cases the use of low quality water is restricted to crops that do not represent a health risk, such as cotton, as produce may be used after processing. Many additional examples of the reuse of wastewater may be found in other regions of the world with regard to the type of water reused for irrigation, crops, and agricultural area.

Both the use of treated and non-treated wastewater entail risks associated with the presence of pathogens. Among these pathogens, helminths (worms), which are responsible for helminthiasis diseases, are of particular concern. Where polluted water is used for agricultural irrigation, helminthiasis are among the main associated diseases that low-income regions face (Keraita et al., 2008, WHO, 2012a). Globally, it is estimated that around 2.5 billion people are affected with helminthiasis (Table 1). These are different types of diseases that result in diarrhea, severe problems of undernourishment, and anemia, mostly in children between 5 and 15 years of age, affecting their quality of life and physical and mental development (Mascarini-Serra, 2011, WHO, 2012a and WHO, 2012b, Vercruysse et al., 2012, Strunz, 2014). This effect in the quality of life of population infected may be figured out with, for example, the 2013 estimation of years lived with disability (YLDs) for anemia, which accounted for 1′004,000 years caused by hookworm disease, and it was of 671,000 years caused by Schistosomiasis (Vos et al., 2015). In fact, the analysis of these estimations indicated that ascariasis was one of the eight causes that affected more than 10% of world population in 2013, considering the mean prevalence of chronic sequelae, for longer than 3 months, of 804′370,000 cases. This figure is twice the prevalence of trichuriasis and hookworm disease, and four times the prevalence of schistosomiasis (Vos et al., 2015). While many helminths are transmitted via contact with contaminated soil (e.g. *Ascaris lumbricoides, Trichuris trichiura* and hookworms), others require the presence of intermediate hosts (e.g. freshwater snails in case of schistosomiasis). Nonetheless, most helminthiasis are transmitted by the eggs through a human-water-soil-crop-human pathway. These eggs are highly infectious (commonly, one egg suffices), highly persistent in the environment, and very resistant to conventional disinfection/inactivation processes (WHO, 2012a, WHO, 2012b, Pullan et al., 2014, Strunz, 2014).

To safely reuse wastewater for irrigation, there is a need to provide a reliable treatment that, among other things, reduces the content of helminth eggs to the levels set in national standards or international criteria. For instance, the World Health Organization suggests that a limit of <1 helminth egg per liter (HE/L) in wastewater makes it safe to reuse for irrigation (WHO, 2006). Based on these guidelines, many countries have set standards for helminth eggs, including Brazil, Colombia, Costa Rica, Chile, Israel, Jordan, Mexico, Saudi Arabia, and Tunisia. Also following WHO (2006) guidelines, restrictions are recommended in terms of the type of crops to be irrigated, irrigation methods, and other intervention measures to manage risks. However, to enforce all these standards it is necessary to measure the helminth egg content in the wastewater (treated or not) intended for irrigation.

In order to quantify the helminth eggs in wastewater, the analytical procedure is based on their identification and enumeration. However, the current methodologies are not always effective for identification as experienced technicians are required, and therefore results are often neither accurate nor reliable. The currently available analytical procedures have two steps. The first step involves separating as many eggs as possible from other particles in the wastewater. This separation aims to concentrate the eggs from samples larger than 5 L to a relatively small sample (1–2 mL) that may be then observed under the microscope. This concentrated sediment still contains many other types of particles and only a properly trained technician is able to discriminate such particles from the eggs. Also, this technician has to visually identify the different species of helminth eggs, potentially present in different life stages, which may be contained in the sample to enumerate them one by one. This second step (visual identification) is critical and constitutes the main source of error and uncertainty in the methodology. This is a problem when samples have a high content of helminth eggs because the discrimination process is so tedious. In addition, when wastewater has a low content of helminth eggs (such as samples that need to meet the standards) they may be difficult to detect. The whole procedure is a very time consuming process, as in addition to the time needed to prepare the sample for microscopic observation or “reading” (around 2 days), the identification step takes approximately 2–5 h for samples with high suspended solids content (TSS) (wastewater sludge, biosolids, or excreta) or 30–60 min for clean samples (less than 15 mg/L TSS). This becomes impractical when several samples are to be analyzed. In short, the challenge for the analysis is to detect, correctly identify, and enumerate helminth eggs in samples that, even after processing, contain many impurities which render this difficult.

To overcome these problems, the aim of this research was to develop digital imaging system to rapidly and reliably identify and quantify seven species of helminth eggs, commonly found in wastewater, at their different stages of development.

## 2 Materials and methods

To develop the system, several steps were followed. These included selecting the species of helminth eggs to be considered by the system, setting up a reference digital image data base for calibration, the selection of appropriate helminth egg properties and the design of the associated algorithms to build the system (system development), and a validation step. The system was developed stepwise in Matlab (MathWorks^®^), incorporating different image processing tools and pattern recognition algorithms as described below, as recommended by different authors (Perona and Malik, 1990, Belanche-Muñoz and Blanch, 2008, Dogantekin et al., 2008, Acvi and Varol, 2009). The system was tested in a personal computer with an Intel^®^ Xeon^®^ processor and 8 GB of RAM memory.

### 2.1 Selection of helminth eggs species

**Fig. 1:**
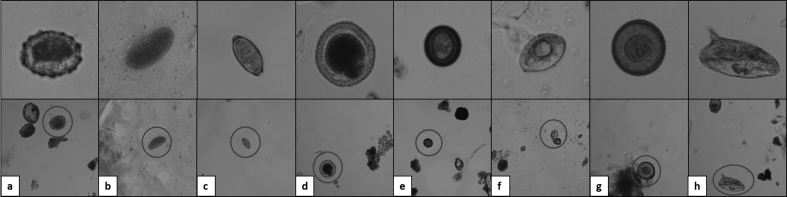
Isolated (top) and whole microscopic images (bottom) of the selected helminth egg species: (a) fertile *Ascaris lumbricoides*, (b) unfertile *Ascaris lumbricoides*, (c) *Trichuris trichiura, (d) Toxocara canis*, (e) *Taenia saginata, (f) Hymenolepis nana,* (g) *Hymenolepis diminuta and (*h) *Schistosoma mansoni*.

The system was validated for seven species selected based on their medical importance and worldwide ubiquity. These were *Ascaris lumbricoides* -fertile and unfertile eggs-, *Trichuris trichiura, Toxocara canis, Taenia saginata, Hymenolepis nana, Hymenolepis diminuta,* and *Schistosoma mansoni* (Fig. 1). At the beginning of the research (system Version 1) only four species were analyzed while for the other versions of the system (Versions 2 and 3) seven species were considered (Table 2). *Hymenolepis* was selected considering the difficulty to correctly identify these species even for trained technicians, while *Schistosoma* was chosen because it is a widespread genus relevant in the public health field, especially in Africa and South America.

### 2.2 Helminth egg image database

A total of 720 high quality images of identified helminth eggs, including the selected species, were collected in a database. These images were obtained from samples of wastewater, sludge, and excreta processed at the laboratory.

Images were taken using a Carl Zeiss AxioLab A1 optical microscope and an Imaging Development Systems UI-1480LE-C-HQ USB2 color camera. To collect homogenous images, all photographs were acquired using 2560 × 1920 pixel resolution without compression.

The images included different stages of egg development, including larval and non-larval eggs, as well as texture and morphological variations within species that may be visually differentiated (e.g. size, number of cells or location of the nucleus; type of membrane: mamillated or non-mamillated).

The species identification at this step was performed by experienced staff in laboratories of the Treatment and Reuse Group (Institute of Engineering UNAM), the Experimental Medicine Unit of the National Medical Center (*Centro Médico Nacional Siglo XXI),* the Inmunoparasitology Laboratory of the Institute of Biology (IBUNAM), and the Parasitology Institute of the Academy of Sciences of the Czech Republic (Dr. František Moravec).

### 2.3 Development of the system for digital identification

Several digital image processing techniques were tested to build the system. As mentioned previously, three different versions of the system were produced.

Version 1 included the following image processing steps: low pass filtering (smoothing), contrast adjustment, object detection, and object labelling. The object detection step utilized a median filter and the corresponding algorithm contained an adaptive histogram equalization, edge detection, distance transform, and watershed algorithm. Once an object in the sample was detected, it was classified according to its shape (area, perimeter, and eccentricity) and texture properties (energy, mean gray level, contrast, correlation, and homogeneity). After training the system using the database images, the system was capable of identifying any object in the image by using a nearest neighbor classifier with the *Mahalanobis* distance metric (Xiang et al., 2008).

**Fig. 2:**
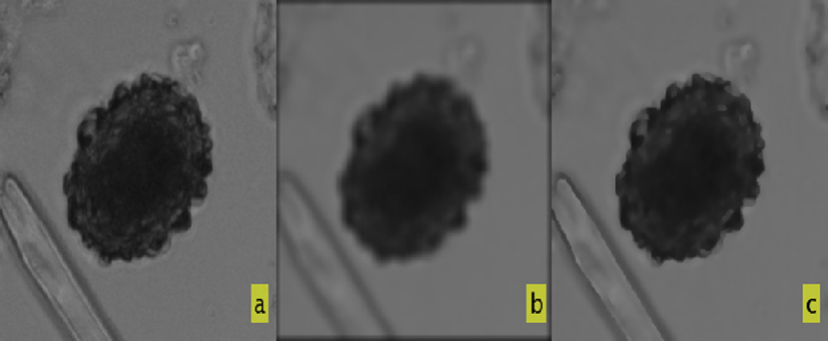
Version 1 and Version 2 Filter comparison: (a) Original *Ascaris* fertile egg image; (b) Median filtered image (Version 1); and (c) Anisotropic diffusion filtered image (Version 2).

Subsequently, two modifications were made to improve the system (Version 2). Firstly, the median filter was replaced with an anisotropic diffusion filter (Perona and Malik, 1990), in order to increase the definition and the detection of the borders of each object, while smoothing the rest of the image (Fig. 2). Secondly, a Gaussian derivative function was applied to further improve the details definition in the outer shell of the eggs. In this way, the algorithm presented the information of the detected image in a binarized mode.

Since the results of the aforementioned Version were not as good as expected, Version 3 of the system was developed. Three additional steps were included: (a) segmentation, (b) the filtered distance transform and watershed algorithm, and (c) Morphological filtering.

**Fig. 3:**
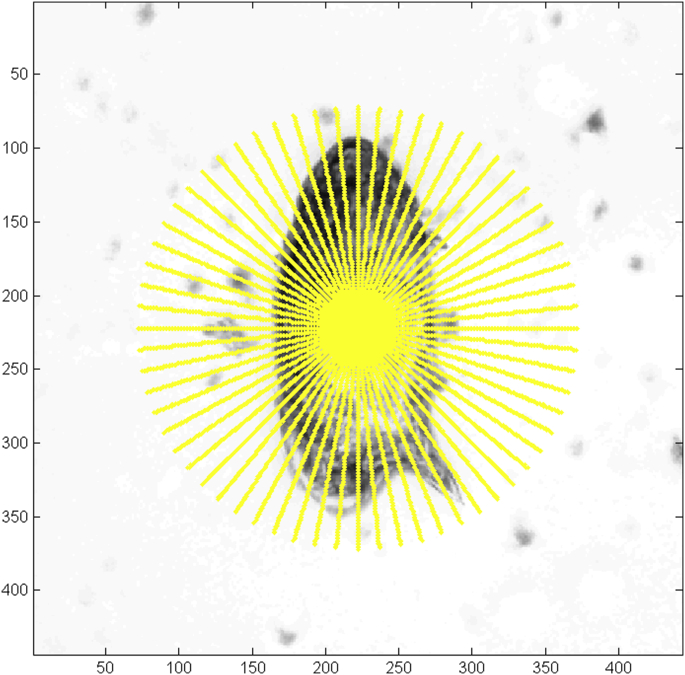
*Schistosoma mansoni* egg: segmentation procedure with 64 gray level profile series for different gray-scale levels.

The segmentation step was based on a gray-scale profile that renders 64 gray level profiles from the center of the object as a starting point, and up to 1.5 times the size of its main axis, which is useful to identify the shape of the helminth egg and thus to distinguish genera and species (Fig. 3).

**Fig. 4:**
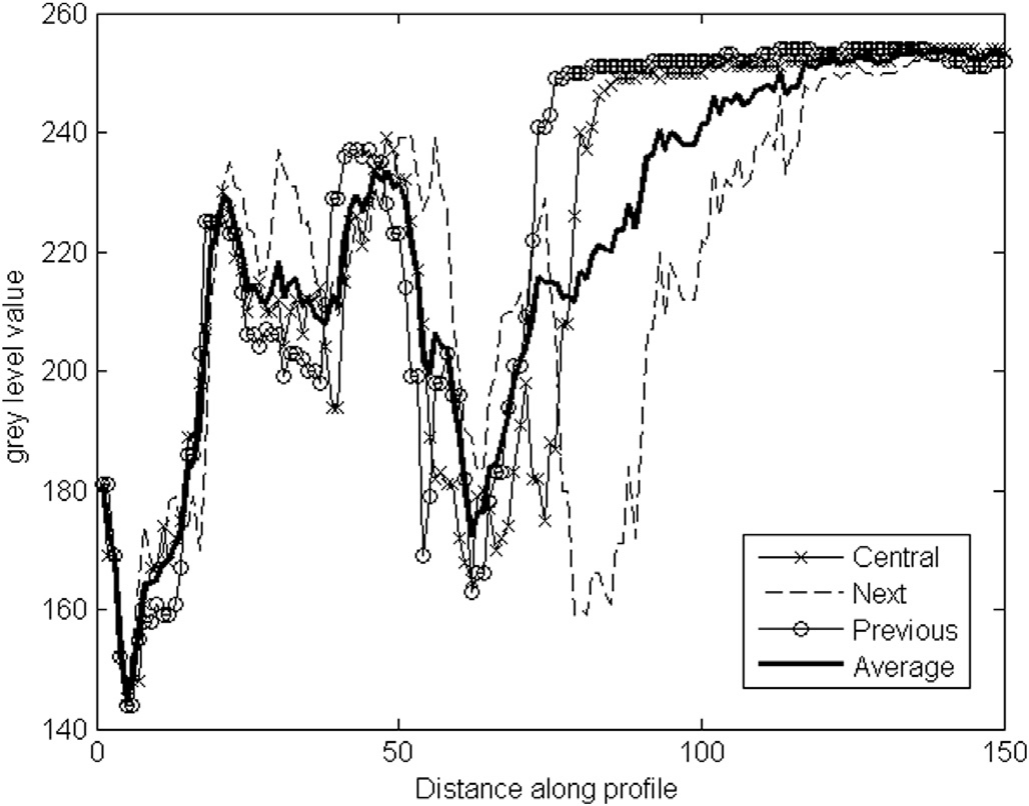
Mean grey profiles: “Average”, “Central” $P_{cent}$, “Next” $P_{next}$, and “Previous”. $P_{pre}$.

Subsequently, an average profile for each of the 64 (5.625° each) was obtained. This was calculated by using the current profile, as the center, as well as the next and the preceding profiles (in counterclockwise order) in the segmented image. Fig. 4 shows the mean gray-scale levels for the previous ($P_{pre}$), central ($P_{cent}$) and next profiles ($P_{next}$).

(1) $$P_{average}=\frac{P_{pre}+P_{cent}+P_{next}}{3}$$

To determine the object border, the mean gray value of the image background is defined. Once the average profile has been calculated, the mean gray level of the image background is estimated considering the external half of the image profile that corresponds to the major axis to the end of the gray profile, i.e.:

(2) $${\text{Background}}_{\text{mean}}=\frac{1}{N}\sum_{i=major\;axis}^{N}P_{average}\left(i\right)$$

To separate the object from the background, the gray level threshold is calculated as follows:

(3) $$\text{Threshold}\;=\;{\text{Background}}_{\text{mean}}-\;Std_{\text{Background}}\;$$

With the background standard deviation given by:

(4) $$Std_{\text{Background}}=\frac{1}{N}\sum_{i=major\;axis}^{N}{\left(P_{average}\left(i\right)-{\text{Background}}_{\text{mean}}\right)}^{2}$$

**Fig. 5:**
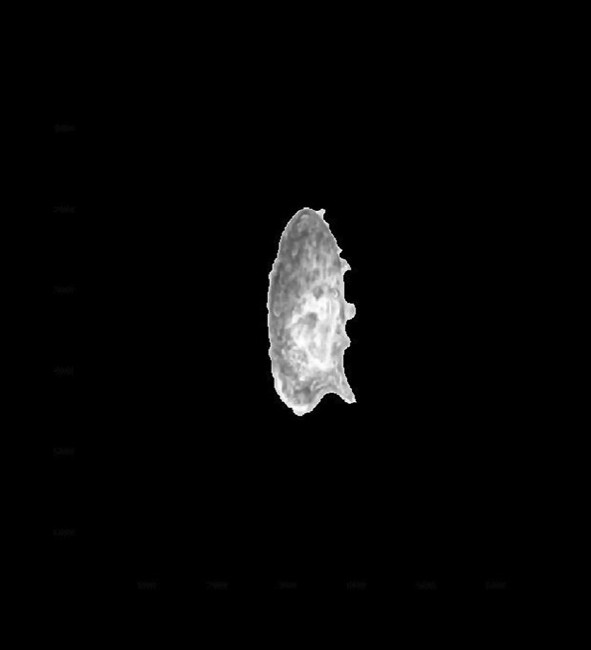
Final result after applying the threshold on the gray-scale profile, obtaining the area to be taken as the *Schistosoma mansoni* egg structure.

In this way all gray level values lower than the threshold will be considered by the system as part of an object (Fig. 5).

The application of a distance field transform and a watershed algorithm was to separate different overlapping objects and to improve the identification of the species (Arambula et al., 2005).

In order to reduce the number of objects processed by the classifier, two morphological filters were applied, the first with a range of compactness index and the second with a range of areas for each object. In this way the objects were classified into two groups: those identified as helminth eggs, and all of the remaining objects, such as bubbles, pollen, pine spores, vegetable waste, yeasts, fat, cell debris, bacteria flocs, crystals, etc. These are objects that may be frequently counted as helminth eggs by the technicians.

**Fig. 6:**
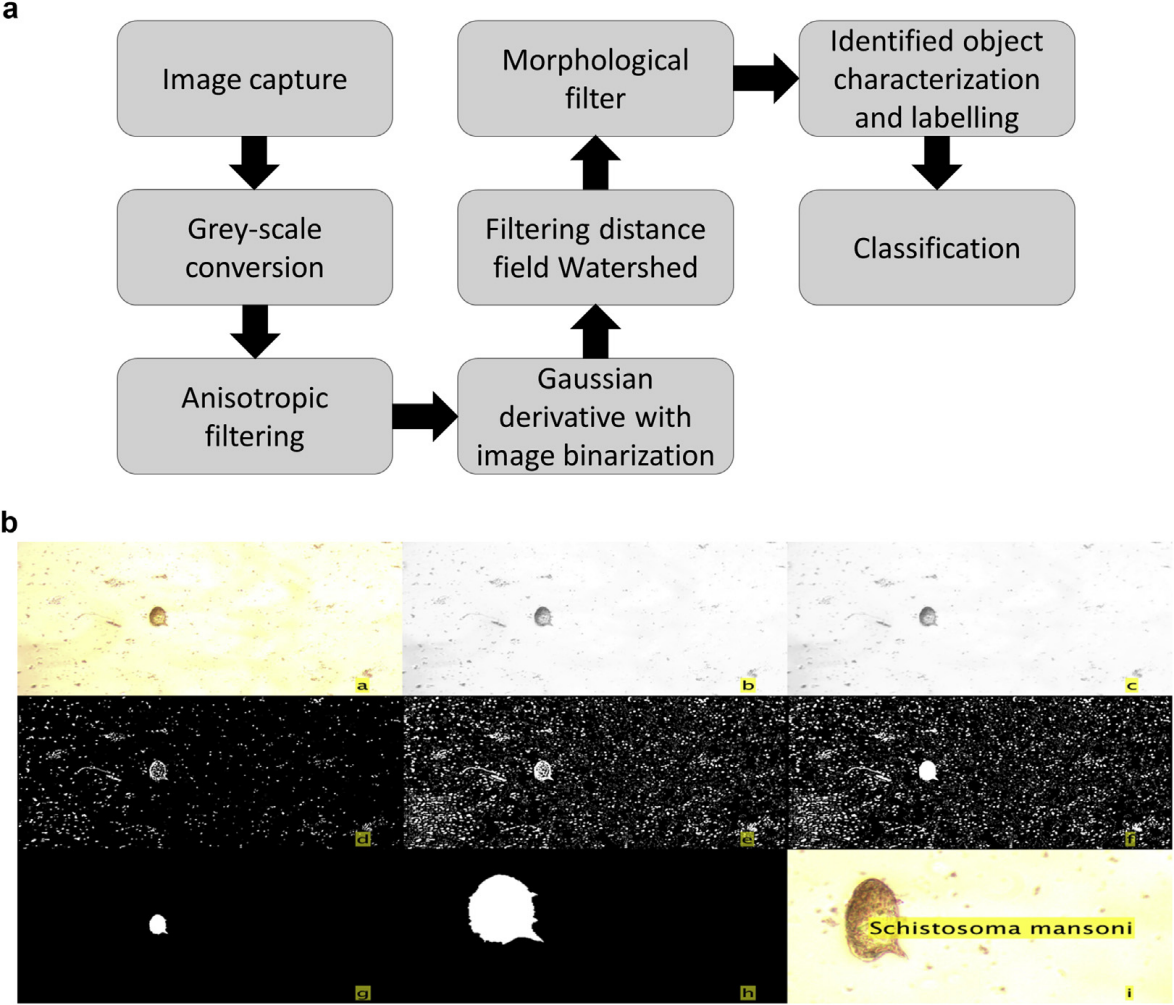
a.Sequence performed by the helminth eggs identification system. b. Example of the digital image processing sequence to identify a Schistosoma mansoni egg: a) Captured image, b) Grey-scale image, c) Image after anisotropic filtering, d-f) Image binarization using a Gaussian derivate function and morphological operation, g) Image after filtering objects by perimeter compactness and minimum and maximum area, h) Zoom of the final image of the egg to be classified i) Object labeling.

The system continues by only using the first group of objects (helminth eggs), to classify them by species based on their different properties as obtained from the database. For this purpose, a naïve bayesian classifier (Belanche-Muñoz and Blanch, 2008) was used to assess the area, diameter, eccentricity, compactness, entropy, edge roughness, and size of minor/major axis properties for each egg. Fig. 6a presents the complete sequence of system Version 3 while Fig. 6b shows an example of the image outcome of such sequence, leading in this case to the identification of a *Schistosoma mansoni* egg.

### 2.4 Validation of the algorithm

Suspended particles are the main interference in the identification and quantification of helminth eggs. To validate the system, wastewater samples with different contents of total suspended solids (TSS) were used to represent the diverse types that may be analyzed in the laboratory (see Appendix Table). Class I corresponded to a low content of TSS (less than 15 mg/L) such as samples from effluents of secondary treatment processes. Class II, with 15–150 mg/L of TSS represented partially treated wastewater and finally, Class III corresponded to samples simulating raw wastewater with a TSS above 150 mg/L (Metcalf and Eddy Inc, 1991, WHO, 2006). Version 1 was validated using Class I samples and four species of helminth eggs (*Ascaris lumbricoides* -fertile and unfertile eggs-*, Trichuris trichiura, Toxocara canis,* and *Taenia saginata*). Validation was performed with 360 images of helminth eggs.

Versions 2 and 3 were validated for the same four species used for Version 1 plus three additional species (*Hymenolepis nana, Hymenolepis diminuta*, and *Schistosoma mansoni)* and for the three Classes of wastewater. The validation tests conducted along this work are summarized in Table 2.

Validation of the system was made by controlling the helminth egg content and the water quality of the sample (TSS content). For this purpose, 5 L of the different classes of wastewater were processed using the US EPA methodology (Yanko, 1987). This methodology comprises four main steps: (1) washing, (2) filtering, (3) floating, and (4) settling, all of them applied to concentrate the eggs into a small suspension volume. The flotation step is used to separate the helminths egg from other detritus, as far as possible, and is performed by adding 150 mL of a saturated chemical solution of zinc sulfate with a specific gravity of 1.3. The supernatant is then centrifuged at 600 g (Maya et al., 2012) and the sediment is recovered. Thirty eggs of each selected species were added to this sediment and it was transferred into a 1000 μL Sedgwick Rafter chamber with 50 × 20 mm dimensions and a 1 mm depth (Thomas Scientific, Swedesboro, New Jersey). The results from the standard visual microscope identification and counting procedure were compared with the system performance. The former was carried out by a team of four technicians working in parallel and using the same image that was analyzed by the system.

To assess the proficiency of the system, two parameters were used: sensitivity and specificity. Sensitivity is defined as follows:

(5) $$Se\;=\;\frac{Tp}{\left(Tp\;+\;Fn\right)\;}\;\;$$

where *Tp* is the number of true positive results, i.e., when the system identified the species of the egg as identified by the technicians, *Fn* is the number of false negatives, i.e. the number of eggs that were identified by the technicians but that the system was unable to identify.

Specificity *(Sp)* refers to the percentage of true negatives as follows:

(6) $$Sp\;=\frac{\;Tn}{\left(Tn\;+\;Fp\right)}\;\;$$

where, *Tn* is the number of true negatives provided by the system, i.e., the number of objects different from helminth eggs that were correctly identified, and *Fp*, the number of false positives provided by the system and defined as the number of objects that were incorrectly identified as helminth eggs.

## 3 Results and discussion

### 3.1 System version 1

From the analysis of 360 samples, the mean sensitivity (true positive fraction) for the four helminth eggs species tested was 85%. By species, the lowest sensitivity obtained was for fertile *Ascaris lumbricoides* eggs (66%), followed by *Taenia saginata* eggs (80%), while for unfertile *Ascaris lumbricoides*, *Toxocara canis,* and *Trichuris trichiura* eggs sensitivity was equal and slightly higher (86%). The lower sensitivity for *Ascaris* was explained because for all of the physical characteristics selected to identify the eggs, this species displayed a high variability. In addition, mean specificity was 88%. Even though the system was capable of successfully discriminating the four most common species of helminth eggs from other objects, the results were not as good as desired. This is because an accurate identification of *Ascaris lumbricoides* is crucial since it is the most common species (60%–80% of eggs identified; USEPA, 1992, Capizzi-Banas et al., 2004, Lustigman et al., 2012, WHO, 2012a). A higher sensitivity is also required to reliably measure values as low as 1 helminth egg/L for treated wastewater. Thus, it was concluded that changes were needed to improve the definition of the boundaries of the eggs, to take advantage of additional morphological differences among the species, and also to increase the sensitivity of the system when using water samples with higher suspended solids content which are more difficult to analyze.

### 3.2 System version 2

Version 2 of the system was developed to improve both specificity and sensitivity. This was achieved by measuring additional shape and texture properties for each object (eccentricity, enthropy, mean gray level, contrast, and homogeneity). Thanks to these modifications it was also possible to include more species (*Hymenolepis nana, Hymenolepis diminuta,* and *Schistosoma mansoni*), and to analyze samples with higher content of suspended solids (class II water with 15–150 mg/l TSS). With these improvements, Version 2 increased the system detection capability. For Class I and Class II water samples, both the sensitivity and specificity were similar, despite the different content of suspended solids (Table 3). While the average specificity was significantly higher than the one obtained for Version 1 (more than 10% higher), average sensitivity did not improve, partly due to the introduction of three new species to the sensitivity function. However, the sensitivity for *Ascaris lumbricoides,* in particular, was considerably improved (79%). In other words, the system gained in its ability to discriminate objects that are not eggs but its average capability to correctly identify helminth eggs remained the same, a situation commonly found in pattern recognition (De Sa, 2012). These results highlighted the need to look for an option to increase the sensitivity of the system.

### 3.3 System version 3

**Fig. 7:**
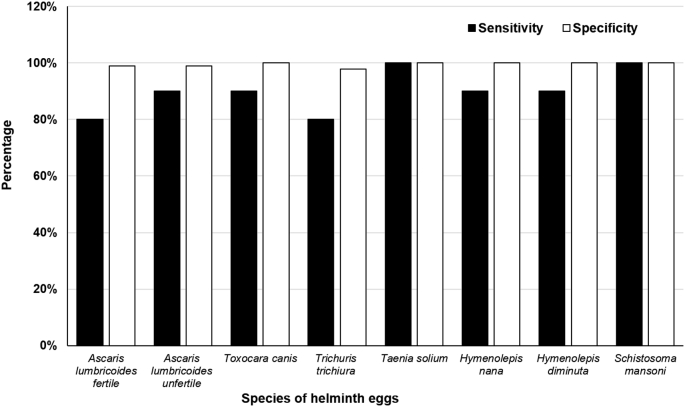
Sensitivity and specificity for the different species tested on Class I wastewater with system Version 3.

**Fig. 8:**
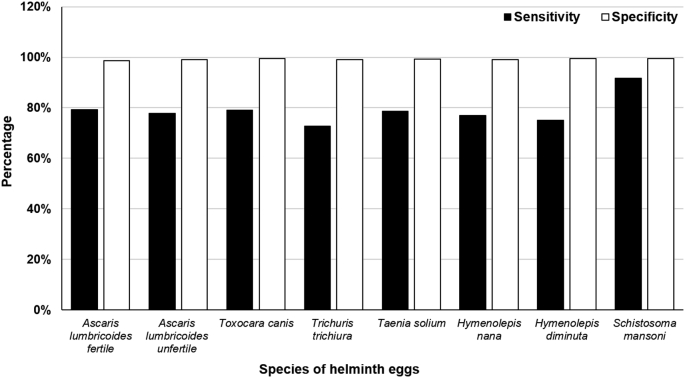
Sensitivity and specificity for the different species tested on Class II wastewater with system Version 3.

This final version of the system was tested for the three classes of wastewater. Fig. 7, Fig. 8 present the validation results. The mean sensitivity for Class I samples increased to 90% and the specificity remained high (99%), while for Class II these numbers were 80% and 99%, respectively. These results were significantly better for all the species tested. Nevertheless, Version 3 exhibited limitations with samples with very high solids contents (Class III, equivalent to raw wastewater), for which sensitivity was found to be considerably lower (<15%) than for Class I or II samples. This is due to the large amount of debris/objects interfering with the precise identification of eggs. To overcome this situation, when wastewater samples with high TSS are to be analyzed, it is recommended to dilute the concentrated sediment with tap water at a 1/1 or 1/2 ratio (v/v) in order to obtain the maximum values registered for the sensitivity and specificity in this study.

The results obtained with system Version 3 represent a significant advance in the analysis of helminth eggs. Yang et al., 2001 have reported the use of a system but with an application limited to clinical samples and only a few helminth eggs species. Their results show a detection rate (sensitivity) of 84% while values for specificity have not been reported. Other studies (Acvi and Varol, 2009; and Dogantekin et al., 2008) have performed the classification/identification of eggs based on only three characteristics that are not useful to analyze helminth eggs contained in wastewater samples, since the system is not capable of identifying different species surrounded by debris commonly found in wastewater, sludge or excreta. In addition, those particular pieces of system based their classification on a Multi-Class Classifier (MCSVM: Multi-Class Support Vector Machine) that renders the process very slow, while the one proposed in this work uses a naïve Bayesian classifier which demands a smaller processing time. All of these differences make the system developed in the current study a versatile and robust tool for identifying parasite eggs in several types of samples. Future applications may include training of parasitologists, supporting field testing by non qualified parasitologists in epidemiological studies, or even the use of hand-held devices (e.g. smartphones) for identification and quantification of helminth eggs.

In summary, the system developed reliably identifies seven species of helminth eggs, reducing uncertainty in the results as well as the time and cost required for quantification (less than 0.5–2 h compared to up to 2–5 h of identification with the traditional technique for samples with high solids content). In addition, the system might be useful as a remote analysis system for communities that require helminth egg quantification and identification but do not have highly trained personnel to carry out direct observation using a microscope. The preparation of samples, being a simple procedure using basic equipment, may be performed in laboratories worldwide.

An application for a patent protecting the developed protocol has been submitted (MX/a/2013/01 0641).

## 4 Conclusions

The system developed here is able to efficiently identify and quantity helminth eggs in commonly found wastewater samples with consistent results. i.e., *Ascaris lumbricoides* -fertile or unfertile-, *Trichuris trichiura*, *Toxocara canis, Taenia saginata, Hymenolepis nana, Hymenolepis diminuta,* and *Schistosoma mansoni*. Even though some helminths are transmitted through larvae, at this stage the system developed focuses on helminth eggs detection. The main advantage of using this system is that it does not require highly trained personnel (i.e. expert parasitologists). Additionally, results are obtained with the same reliability, sensitivity and specificity, allowing the comparison of data among countries/regions. This is a consideration that has been limited thus far, since data on helminth eggs are variable depending on analytical capacity on a regional basis. The use of this system is expected to reduce identification costs, and to bring the option to promptly and reliably detect helminth eggs to a much wider community. Moreover, the system demonstrated the following advantages:

• It provides a uniform criteria for helminth egg identification which reduces process uncertainty.
• The flexibility of the image processing tools allows an increase in its identification abilities in terms of water quality of the samples, and the number of species that could be included in the identification database.
• It confers better species classification, due to morphologic and texture characteristics.
• It results in a reduction in the time required for identification and quantification.

Based on these results, it is important to select characteristics which appropriately describe the different types of helminth eggs commonly found in environmental samples to develop a suitable and reliable system to identify and quantify them. As shown in this research this has to be performed stepwise and therefore in order to add additional species other properties and algorithms might need to be added. The selection of more than 15 parameters (most of them described above as patent is pending) is the strength of the software in the sense that it prevents the eggs to be confused with other objects. This also ensures that even for similar species, some of those parameters will differ enough to be correctly classified. Also, it is expected that further steps in the system's development will increase its current capabilities and potential, including the addition of further parasites such as protozoa (e.g. *Cryptosporidium* spp., *Giardia intestinalis,* and *Entamoeba histolytica*). Moreover, future implementations of the software may use open-source libraries to allow the widespread use of these tool, especially in developing countries.

## Figures and Tables

**Table:** 

Quality	Type of process that produce the water	TSS (mg/L)	Typical helminth eggs content (HE/L)	Equivalent treatment processes	TSS (mg/L)
Class I	Tertiary treatment: activated sludge + sedimentation + filtration	0.5–0.97	0.0 – <0.2	Tertiary treatment: coagulation/flocculation, high-rate granular or slow-rate sand filtration, dual-media filtration, membrane filtration.	<15
Effluent of physicochemical process + filtration	1.0–3.0	0.0–1.0
Secondary treatment: activated sludge + secondary sedimentation	3 – <15	0.4–1.0	Activated sludge + secondary sedimentation; trickling filters + secondary sedimentation; aerated lagoon + settling pond
Class II	Effluent of physicochemical processes	>15 – 40	1.2–2.0	Advanced primary treatment: coagulation/flocculation + sedimentation	>15–100
Primary wastewater treatment	>40 – 150	8.0–15	Low-rate biological processes: waste stabilization ponds, wastewater storage and treatment reservoirs, constructed wetlands. High-rate processes (Primary treatment): primary sedimentation; chemically enhanced primary treatment; up flow anaerobic sludge blanket reactors.	>100–150
Class III	Untreated municipal wastewater	240–763	13–70	Untreated municipal wastewater	>150–1200

Source: Metcalf and Eddy Inc, 1991, WHO, 2006.

**Table 1:** Helminth species and world helminthiasis reported in different regions.

Helminth species	Common name	Prevalence (million inhabitants)	Regional presence
**Nematoda**			
*Ascaris lumbricoides*	Roundworm	819	Many regions of South-east Asia, Africa, and Central and South America^a^
*Ancylostoma duodenale*	Hookworms	439	Tropical and subtropical countries (Sub-Saharan Africa)^a^^,^^b^
*Necator americanus*
S*trongyloides stercoralis*	370
*Trichuris trichiura*	Whipworm	465	Moist, warm, tropical regions of Asia, Africa, Central and South America, and the Caribbean islands^a^
*Trichostrongylus orientalis*	Roundworm	Several	Mainly rural communities in Asia^c^
**Cestoda**			
*Hymenolepis nana*	Dwarf tapeworm	50	Most occurrences in areas which lack adequate sanitation and can be found around the world in South America, Southeast Asia, West Africa and East Africa; and in areas of the tropics and subtropics and some areas of Southern and Eastern Europe and the United States of America d^,^^e^
*Taenia solium*	Pork tapeworm	50
**Trematoda**			
*Schistosoma mansoni*	Blood fluke	207	Tropical and subtropical regions^f^
*Clonorchis sinensis* *Echinostoma* spp *Fasciola gigantica* *Fasciola hepatica* *Fasciolopsis buski* *Heterophyes* spp. *Metagonimus* spp. *Opisthorchis felineus* *Opisthorchis viverrini* *Paragonimus* spp.	Food-borne trematods	56	Largely in Southern and Eastern Asia but also in Central and Eastern Europe^g^
Other groups		100	Worldwide a^,^^h^
Total		Over 2.5 billion infections worldwide	

With information from:

a Pullan et al., 2014.

b Bisoffi et al., 2013.

c UN, 2003.

d Eddi et al., 2003.

e Torgerson and Macpherson, 2011.

f Steinmann et al., 2006.

g Fürst et al., 2012.

h Lustigman et al., 2012.

**Table 2:** Summary of the species and classes of water tested in each system version.

	Class I water	Class II water	Class III water	Species detected
System Version 1	**X**			Fertile and unfertile *Ascaris lumbricoides*, *Trichuris Trichura*, *Toxocara canis*, *Taenia saginata*
System Version 2	**X**	**X**	**X**	Species of system V.1 + *Hymenolepis nana*, *Hymenolepis diminuta*, *Schistosoma mansoni*
System Version 3	**X**	**X**	**X**	Species of system V.1 + *Hymenolepis nana*, *Hymenolepis diminuta*, *Schistosoma mansoni*

**Table 3:** Validation results (mean sensitivity/specificity) for each system version.

	Class I water		Class II water		Class III water	
Sensitivity	Specificity	Sensitivity	Specificity	Sensitivity	Specificity
Version 1	85%	88%	NP	NP	NP	NP
Version 2	83%	99%	80%	98%	NP	NP
Version 3	90%	99%	80%	99%	15%	1.0%

NP: Not performed.
